# iPSC-derived type IV collagen α5-expressing kidney organoids model Alport syndrome

**DOI:** 10.1038/s42003-023-05203-4

**Published:** 2023-09-28

**Authors:** Ryuichiro Hirayama, Kosuke Toyohara, Kei Watanabe, Takeya Otsuki, Toshikazu Araoka, Shin-Ichi Mae, Tomoko Horinouchi, Tomohiko Yamamura, Keisuke Okita, Akitsu Hotta, Kazumoto Iijima, Kandai Nozu, Kenji Osafune

**Affiliations:** 1 Center for iPS Cell Research and Application (CiRA), Kyoto University, Kyoto, 606-8507 Japan; 2 Taisho Pharmaceutical Co., Ltd., Saitama, 331-9530 Japan; 3 Department of Pediatrics, Kobe University Graduate School of Medicine, Hyogo, 650-0017 Japan; 4 Hyogo Prefectural Kobe Children’s Hospital, Hyogo, 650-0047 Japan; 5 Department of Advanced Pediatric Medicine, Kobe University Graduate School of Medicine, Hyogo, 650-0017 Japan

**Keywords:** Alport syndrome, Induced pluripotent stem cells

## Abstract

Alport syndrome (AS) is a hereditary glomerulonephritis caused by *COL4A3*, *COL4A4* or *COL4A5* gene mutations and characterized by abnormalities of glomerular basement membranes (GBMs). Due to a lack of curative treatments, the condition proceeds to end-stage renal disease even in adolescents. Hampering drug discovery is the absence of effective in vitro methods for testing the restoration of normal GBMs. Here, we aimed to develop kidney organoid models from AS patient iPSCs for this purpose. We established iPSC-derived collagen α5(IV)-expressing kidney organoids and confirmed that kidney organoids from *COL4A5* mutation-corrected iPSCs restore collagen α5(IV) protein expression. Importantly, our model recapitulates the differences in collagen composition between iPSC-derived kidney organoids from mild and severe AS cases. Furthermore, we demonstrate that a chemical chaperone, 4-phenyl butyric acid, has the potential to correct GBM abnormalities in kidney organoids showing mild AS phenotypes. This iPSC-derived kidney organoid model will contribute to drug discovery for AS.

## Abstract (web summary)

The development of a kidney organoid model from induced pluripotent stem cells (iPSCs) recapitulates collagen composition from mild and severe cases of Alport syndrome, establishing a kidney organoid model aiding drug discovery.

## Introduction

Alport syndrome (AS) is the second most common hereditary glomerulonephritis that proceeds early in life to end-stage renal disease (ESRD)^1–4^. It is caused by mutations in *COL4A3*, *COL4A4,* or *COL4A5* genes and defects in α3α4α5(IV) heterotrimers that compose glomerular basement membranes (GBMs)^5–7^. Some clinical studies show differences in the disease progression that depends on the mutation types, and there is a correlation between the detection of α5(IV) and clinical symptoms^4,8^. However, the disease mechanisms have not been fully elucidated, and there are no curative treatments^9,10^. Further, the lack of efficient in vitro tools to analyze the collagen composition of GBMs has made it difficult to develop curative therapeutic approaches that correct collagen abnormalities in AS.

Type IV collagen is a main component of basement membranes and functions as heterotrimers: α1α1α2(IV), α3α4α5(IV), or α5α5α6(IV). In particular, α3α4α5(IV) is mainly localized in GBMs and constitutes the glomerular filtration barrier^7,11–13^. In development, mature podocytes switch to produce α3α4α5(IV) from α1α1α2(IV) around the capillary loop stage^11,13–15^. These developmental changes are essential to construct a robust filtration system, and *COL4A5* mutations disrupt normal switching and cause renal dysfunction^11^. Type IV collagens form heterotrimers from the NC1 domain in their C-terminus as a starting point^16^. Thus, mutation types that lead to NC1 domain anomalies or defective trimer formation cause severe AS clinical symptoms. In such cases, no α3/α4/α5(IV) subunits are detected in GBMs^17,18^. In contrast, some mutations do not hamper trimer formation but lead to the formation of misfolding structures. These mutations are correlated with mild AS symptoms^18^.

Previous studies reported the generation of various AS mouse models, such as *Col4a5*^tm1Yseg^ (p. G5X) mice, which exhibit similar phenotypes to humans, including GBM anomalies, proteinuria, and renal fibrosis^19–21^. Although the mouse models have advantages in that renal function can be assessed, it is difficult to validate a direct approach to GBMs for the development of new therapies because of the time required to create and evaluate them. Higher-order human cell evaluation systems are useful considering species differences.

Kidney organoids are self-organizing 3D cellular aggregates that contain nephron-like structures and are differentiated from embryonic stem cells (ESCs) or induced pluripotent stem cells (iPSCs) by mimicking kidney developmental processes^22–25^. In embryonic kidneys, the GBM collagen composition changes from α1α1α2(IV) to α3α4α5(IV) heterotrimers during the transition from the S-shaped stage to capillary loop stage of nephrogenesis^11,12,14,15,26^. Although kidney organoids also develop avascular glomerulus structures containing podocyte-like cells in vitro, the collagen composition of the GBM-like structures is immature^27^. While a recent study revealed that kidney organoids conserve the temporal sequence of the basement assembly^28^, no reports have reproduced AS disease phenotypes using kidney organoids differentiated from patient iPSCs.

In this study, we successfully generated kidney organoids that contain GBM-like structures with α3α4α5(IV) collagen heterotrimers using agitated cultures with an orbital shaker to mature the collagen composition in a time-dependent manner. We established iPSCs from two male X-linked AS (XLAS) patients carrying COL4A5 mutations on the X chromosome, one mild case with a c.1634 G > A (p.G545D) missense mutation in exon 24 and one severe case with a c.1652_53 dupTC (p.T552Sfs*6) nonsense mutation in exon 24 that causes a truncated form of α5(IV) and lack of heterotrimer formation. We repaired the gene mutation in iPSCs from the mild AS patient, which led to the restoration of the collagen α5(IV) protein expression in the induced kidney organoids. Furthermore, we found that kidney organoids from the AS patient iPSCs showed different abnormal collagen expression patterns in the GBM-like structures depending on the disease severity, suggesting that these cells can reproduce the phenotypic differences among AS patients. Finally, we found that a chemical chaperone can potentially normalize misfolded proteins in kidney organoids from the mild AS patient iPSCs but not from the severe AS patient iPSCs, confirming the recovery of α5(IV) antigen conformation on the GBM-like structures. These results suggest that kidney organoids from AS patient iPSCs are a powerful tool for analyzing disease mechanisms and predicting the effectiveness of therapeutic agents for individual AS patients, enabling personalized medicine.

## Results and discussion

### Generation of AS patient iPSCs

**Fig. 1:**
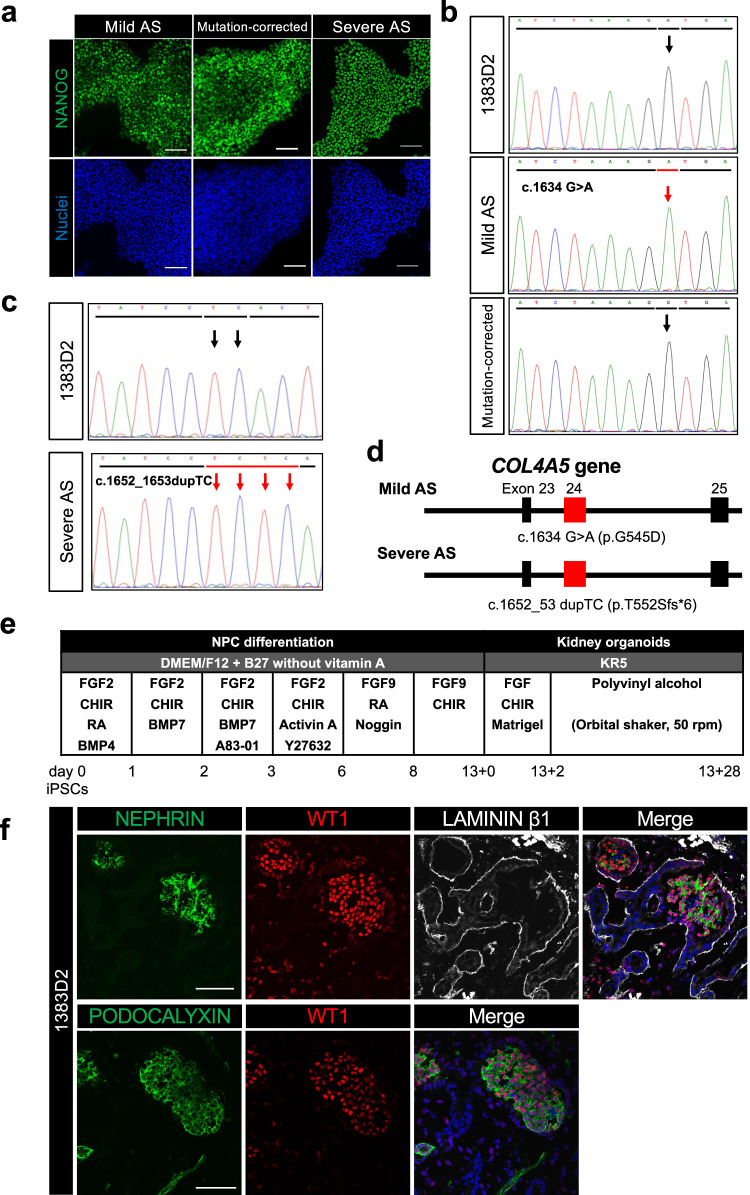
Establishment of AS patient and *COL4A5* gene mutation-corrected iPSCs and generation of kidney organoids. **a** The expression of a pluripotency marker, NANOG, in iPSCs. Scale bars, 100 µm. **b**, **c** The sequencing analysis of exon 24 of *COL4A5* gene in 1383D2 cells, mild AS patient iPSCs, and mutation-corrected mild AS patient iPSCs (**b**) and in 1383D2 cells and severe AS patient iPSCs (**c**). Black and red bars and arrows indicate normal and mutated sequences, respectively. **d** A schematic showing *COL4A5* mutations in mild and severe AS patients. **e** The kidney organoid differentiation protocol. **f** Immunostaining images of 1383D2 cell-derived day 13 + 28 kidney organoids for NEPHRIN and WT1 (podocytes) and LAMININ β1 (GBMs and tubular basement membranes) and for PODOCALYXIN (podocytes) and WT1. Scale bars, 50 µm.

We generated iPSCs from the peripheral blood mononuclear cells (PBMCs) of two male XLAS patients with distinct symptomatic severity using episomal plasmids^29,30^. Although both patients were around 20 years old, the mild patient showed only slight proteinuria and hematuria as a symptom, while the severe patient proceeded to ESRD in his late teens. The mild patient’s mother had received a kidney biopsy test and was found α5(IV) negative. To establish disease models for the development of novel AS therapies by correcting the structural abnormalities of type IV collagen heterotrimers with low-molecular-weight compounds, we corrected the point mutation of the *COL4A5* gene in mild AS patient iPSCs by CRISPR-Cas9 ribonucleoprotein and single-stranded DNA-mediated homologous recombination^31^. We confirmed that all iPSCs used in this study exhibited a normal morphological appearance, normal karyotype, and expression of the pluripotency marker NANOG (Fig. 1a, S1a, b). A genetic analysis of PBMCs revealed that both patients had *COL4A5* mutations in exon 24, with a c.1634 G > A (p.G545D) missense mutation in the mild patient and a c.1652_53 dupTC (p.T552Sfs*6) nonsense mutation in the severe patient. These mutations were preserved in the patient’s iPSCs (Fig. 1b–d).

### AS patient iPSCs differentiate into kidney organoids

To develop a simple culture system suitable for drug screening for AS, we modified our previously reported kidney organoid differentiation method through nephron progenitor cells (NPCs) from iPSCs and adopted floating and agitated cultures with an orbital shaker (Fig. 1e). Culture day 9 cells induced from a healthy control iPSC line (1383D2^22^) were positively stained with NPC markers, including WT1, SALL1, SIX2, PAX2 and HOXD11 (Figure S1c), as previously reported^25^. To quantitatively analyze the induction efficiency of NPCs among four iPSC lines (1383D2, mild AS patient iPSCs, mutation-corrected mild AS patient iPSCs, and severe AS patient iPSCs), we used WT1 and PAX2 as markers of NPCs on days 9 and 13, respectively, but found no significant differences in the differentiation efficiency among the lines (Figures S1d, e). During the 28 days of organoid culture of 1383D2 cells (from days 13 + 0 to 13 + 28), no obvious morphological changes in the bright field microscopy images were observed (Figure S2a). We confirmed that 1383D2-derived kidney organoids generated in the floating and agitated cultures show higher expressions of *NPHS1*, *NPHS2,* and the collagen IV genes *COL4A3*, *COL4A4,* and *COL4A5* than those in the floating and non-agitated cultures, although the differences in *NPHS1* and *COL4A5* expression were not statistically significant (Figures S2b, c). Furthermore, the agitated cultures resulted in a significantly higher rate of PODOCALYXIN^+^ or EpCAM^+^ nephron-constituent cells than the non-agitated cultures (Figures S2d, S3). Although the mechanisms of these benefits of agitated cultures remain unknown, some possibilities including the diffusion-dependent maintenance of nutrients and oxygenation, waste removal, and mechanical stress were reported in cerebral organoids^32,33^.

In 1383D2-derived kidney organoids, the formation of glomerulus-like structures containing NEPHRIN^+^WT1^+^ or PODOCALYXIN^+^WT1^+^ podocyte-like cell clusters demarcated by LAMININ β1 was observed by immunostaining (Fig. 1f). We also confirmed that the kidney organoids from all four iPSC lines contained PODOCALYXIN^+^ podocyte-like cell clusters, EpCAM^+^*Lotus tetragonolobus* lectin (LTL)^+^ or EpCAM^+^CDH6^+^ proximal tubule-like structures and EpCAM^+^*Dolichos biflorus agglutinin* (DBA)^+^ or EpCAM^+^CDH1^+^ distal tubule-like structures (Figures S4a, S4b, S5a). Furthermore, we confirmed the development of nephron-constituent cells in 1383D2-derived day 13 + 28 kidney organoids by transmission electron microscopy (TEM), including podocyte-like cells exhibiting foot processes and surrounded by parietal epithelial cells, proximal tubule-like cells with brush borders and loop of Henle- or distal tubule-like cells that showed fewer microvilli and cilia and the morphology of a flat or cubic epithelium (Figures S5b-f). Immunostaining of a mild AS patient iPSC-derived day 13 + 28 kidney organoid for the vascular endothelial marker CD31 and the podocyte marker WT1 did not show the vascularization of glomerulus-like structures in kidney organoids (Figure S5g).

### Time-dependent GBM maturation in kidney organoids

**Fig. 2:**
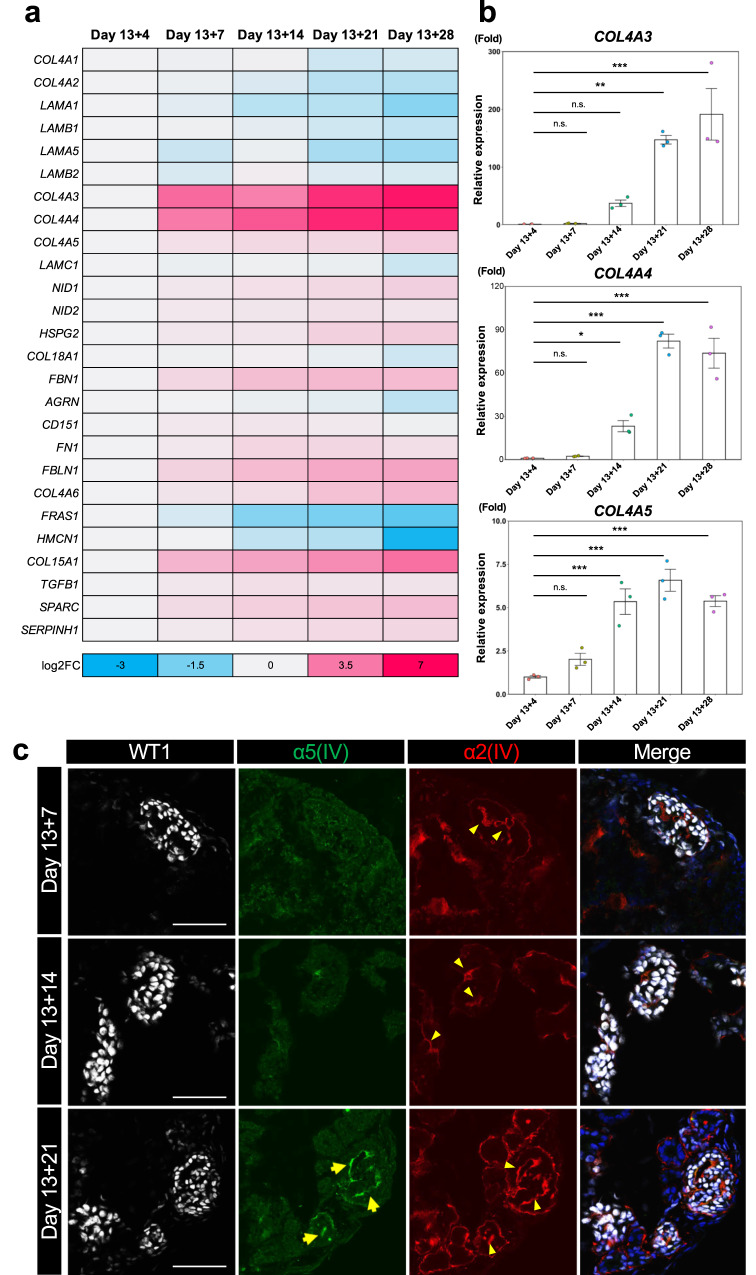
Temporal changes in GBM marker mRNA expression and collagen composition of GBM-like structures. **a** A heatmap showing log2FC gene expression relevant to GBMs in 1383D2 cell-derived kidney organoids on days 13 + 4 to 13 + 28 by RNA-seq analysis. **b** qRT-PCR analysis of 1383D2 cell-derived kidney organoids on days 13 + 4 to 13 + 28 for *COL4A3*, *COL4A4*, and *COL4A5*. The data from three independent experiments are represented as the means ± SEM (*n* = 3). **c** Immunostaining images of glomerulus-like structures in 1383D2 cell-derived kidney organoids on days 13 + 7, 13 + 14, and 13 + 21 for WT1, α5(IV) and α2(IV). Arrows and arrowheads indicate α5(IV)- and α2(IV)-expressing GBM-like structures, respectively. Scale bars, 50 µm. *p < 0.05, **p < 0.01, and ***p < 0.001 by Dunnett’s test, n.s., not statistically significant.

To develop in vitro AS kidney organoid models, the expression of α3α4α5(IV) heterotrimers is necessary. However, we found that the kidney organoids at early culture stages lacked α5(IV) expression in the GBM-like structures (Fig. 2). Therefore, we temporally examined GBM-associated gene expression by RNA sequencing and qRT-PCR analysis in 1383D2-derived kidney organoids on days 13 + 4, 13 + 7, 13 + 14, 13 + 21 and 13 + 28 (Figs. 2a, b). Mature GBM markers, including *COL4A3*, *COL4A4,* and *COL4A5*, were upregulated in a time-dependent manner, whereas early-stage GBM markers, such as *COL4A1*, *COL4A2*, *LAMA1,* and *LAMAB1*, were gradually downregulated. These results indicate a transition in the composition of the collagen IV chains in GBMs from α1α1α2(IV) to α3α4α5(IV). Consistently, immunostaining analysis revealed α5(IV) expression in the GBM-like structures after day 13 + 21 (Fig. 2c, S6a). Thus, long-term agitated culture with an orbital shaker generated kidney organoids containing GBM-like structures with α3α4α5(IV) collagen in vitro, indicating that the organoids can be used to create AS models.

### Kidney organoids containing GBMs with α3α4α5(IV) collagen recapitulate AS phenotypes in collagen composition

**Fig. 3:**
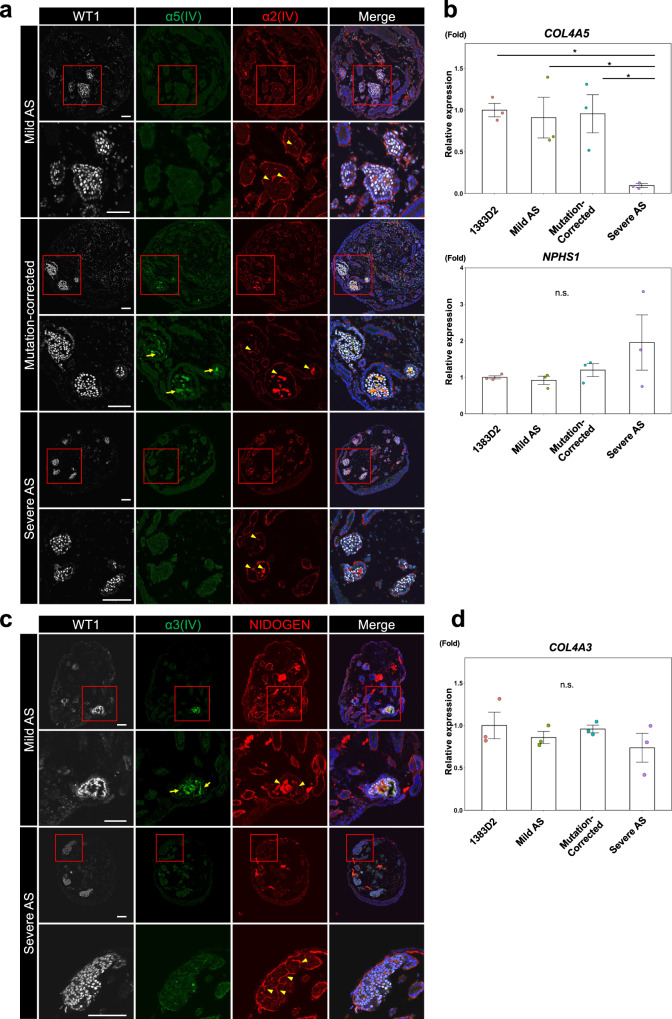
Patient iPSC-derived kidney organoids recapitulate histopathological phenotypes of AS. **a** Immunostaining images of glomerulus-like structures in day 13 + 28 kidney organoids from mild AS patient iPSCs, mutation-corrected mild AS patient iPSCs and severe AS patient iPSCs for WT1, α5(IV) and α2(IV). Magnified images of the red-boxed areas are also shown. Arrows and arrowheads indicate α5(IV)- and α2(IV)-expressing glomerulus-like structures, respectively. Scale bars, 50 µm. **b** qRT-PCR analysis of day 13 + 28 kidney organoids from 1383D2 cells, mild AS patient iPSCs, mutation-corrected mild AS patient iPSCs, and severe AS patient iPSCs for *COL4A5* and *NPHS1*. The data from three independent experiments are represented as the means ± SEM (*n* = 3). **c** Immunostaining images of glomerulus-like structures in day 13 + 28 kidney organoids from mild and severe AS patient iPSCs for WT1, α3(IV), and NIDOGEN. Magnified images of the red-boxed areas are also shown. Arrows and arrowheads indicate α3(IV)- and NIDOGEN-expressing glomerulus-like structures, respectively. Scale bars, 50 µm. **d** qRT-PCR analysis of day 13 + 28 kidney organoids from 1383D2 cells, mild AS patient iPSCs, mutation-corrected mild AS patient iPSCs, and severe AS patient iPSCs for *COL4A3*. The data from three independent experiments are represented as the means ± SEM (*n* = 3). *p < 0.05, by Tukey–Kramer test, n.s. not statistically significant.

We examined whether α5(IV)-expressing kidney organoids have collagen α3α4α5(IV) in their GBM-like structures. Upon confirming α3(IV) protein expression in the NIDOGEN^+^ GBM-like structures in 1383D2 and mutation-corrected mild AS iPSC-derived kidney organoids by immunostaining (Figure S6b), we assumed that the kidney organoids contain GBMs with α3α4α5(IV) collagen chains. Then we tested whether kidney organoids from mild and severe AS patient iPSCs express α5(IV) in the GBM-like structures. Consistent with the clinical histopathology, the expression of α2(IV), but not α5(IV), was observed in kidney organoids on day 13 + 28 from both patient iPSCs, while kidney organoids from mutation-corrected mild AS patient iPSCs expressed α5(IV) protein (Fig. 3a), suggesting that kidney organoids from AS patient iPSCs recapitulate the α5(IV) depletion phenotypes.

Next, we conducted a TEM analysis to clarify whether kidney organoids show GBM ultrastructural abnormalities. We confirmed the GBM ultrastructure on day 13 + 28 organoids from all iPSC lines. However, kidney organoids from both mild and severe AS iPSCs failed to show several GBM ultrastructural abnormalities, such as thickening, splitting, and fragmenting of the lamina densa^34–36^ (Figure S6c). GBM-like structures in the organoids were ~100 nm thick and uniform (arrows in Figure S6c), but some GBM-like structures were divided into two basement membranes, which is consistent with observations of the early developmental stage of GBMs^37, 38^ (arrowheads in Figure S6c). Then, we analyzed the embryonic kidneys of AS model mice that harbor the G5X mutation in *Col4a5* and develop the severe GBM lesions seen in adults, such as lamellation and splitting^19,39,40^. However, we did not detect any GBM ultrastructural abnormalities in E15.5 male *col4a5*^*Y/-*^ mice (Figure S6d). Taken together with the finding that the GBM-like structures of our kidney organoids show isoform switching of type IV collagen (Figs. 2b, c), which occurs around the capillary loop stage in mouse embryonic kidneys^11,12,14,15,26^, we assumed that our kidney organoids are equivalent to around E15.5 mouse embryonic kidneys. These data suggest that our kidney organoids reproduce the switching of type IV collagen isoforms and AS phenotypes in collagen composition but not in ultrastructure.

### Kidney organoid model recapitulates atypical AS phenotypes

*Col4A5* expression is downregulated in AS model mice^19,20^. Consistently, the mRNA expression of *COL4A5* was significantly decreased in our severe AS kidney organoids compared with 1383D2-derived organoids (Fig. 3b). However, *COL4A5* expression did not change in mild AS kidney organoids, and no significant changes were observed in the mRNA expression of a podocyte marker, *NPHS1*, in all kidney organoids from all iPSC lines, although severe AS organoids showed a large batch variation (Fig. 3b). The difference in *COL4A5* expression between mild and severe AS cases was explained by the types of *COL4A5* mutations. Severe AS with a frameshift mutation (T552fs*6) could cause the nonsense-mediated mRNA decay (NMD) of *COL4A5* mRNA, while mild AS with a point mutation (G545D) could not trigger NMD. Further analysis revealed that mild AS kidney organoids expressed α3(IV) protein, but severe AS organoids did not (Figs. 3c, d). Although both α3(IV) and α5(IV) are usually undetectable in typical AS patients, the expression of abnormal collagen proteins has been detected in GBMs by immunofluorescence in atypical cases^18,41,42^. It is known that type IV collagens form heterotrimers from the NC1 domain as a starting point^6,16^. Severe AS organoids showed a significant decrease in *COL4A5* mRNA expression and lack α5(IV) C-terminal regions, including the NC1 domain, because of their mutations, whereas mild AS organoids carried a point mutation that does not significantly affect *COL4A5* mRNA expression levels. These results suggest that α3α4α5(IV) heterotrimers were not present in the GBM-like structures of severe AS kidney organoids, but heterotrimers comprising mutated α5(IV) were expressed in the GBM-like structures of mild AS organoids. Previous clinical studies reported that AS patients with detectable α3(IV) or α5(IV) have mild symptoms, including the later onset of ESRD and hearing loss and fewer ocular abnormalities despite the genetic mutations^8,17,18,42^. Consistently, our results suggest that mild AS iPSCs are capable of differentiating into kidney organoids that recapitulate the clinical hallmark of α3(IV) expression. Thus, our kidney organoids are useful for reproducing individual differences in AS, indicating that iPSC-derived kidney organoids have advantages as a less invasive diagnostic tool for studying α3α4α5(IV) expression over other tests, such as renal biopsy.

### Kidney organoids transplanted into renal subcapsules become vascularized and recapitulate AS phenotypes

**Fig. 4:**
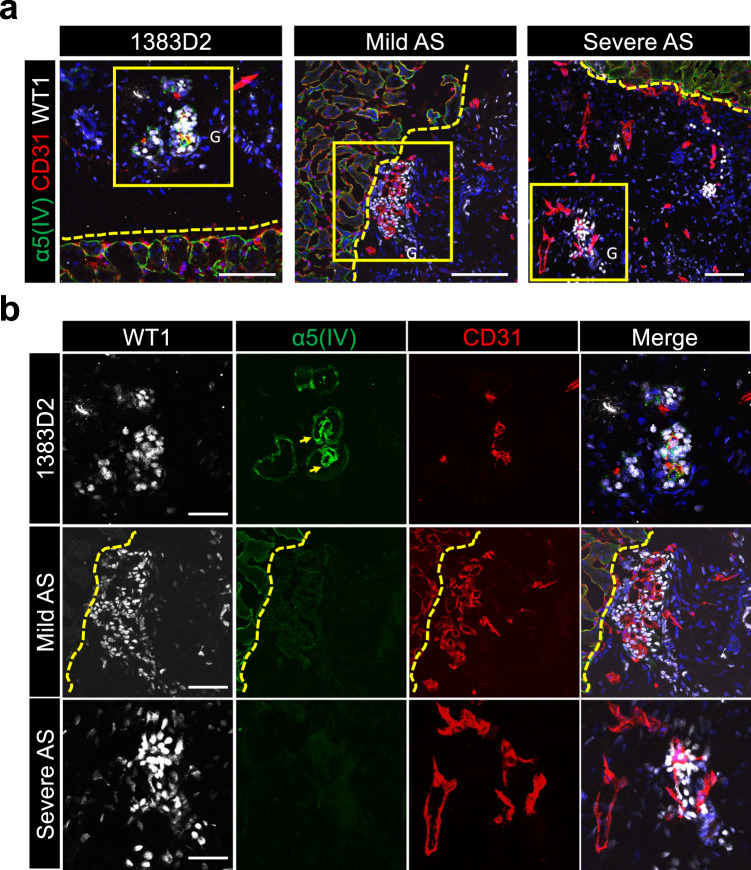
Patient iPSC-derived kidney organoids become vascularized and recapitulate AS phenotypes after transplantation in vivo. **a** Immunostaining images of glomerulus-like structures in day 21 grafts for WT1 (podocytes), α5(IV), and CD31 (mouse endothelial cells). Mouse kidney and grafts from NPCs are divided by the yellow dotted lines. Scale bars, 100 µm. **b** Magnified images of the yellow boxed areas in **a**. Arrows indicate α5(IV)-expressing GBM-like structures. G glomerulus. Scale bars, 50 µm.

In embryonic kidneys, GBMs mature by fusion of the basement membranes of podocytes and vascular endothelial cells^37^. Kidney organoids are vascularized when transplanted into mouse renal subcapsules and develop higher-order renal tissue-like structures^25,43^. Therefore, to more accurately characterize GBM-like structures in kidney organoids, we transplanted day 13 NPCs under the renal capsules of NOD/SCID mice. In the grafts 21 days after transplantation, we found glomerulus-like structures with WT1^+^ podocyte-like cells and CD31^+^ vascular endothelial cells from host mice (Fig. 4a, b). Moreover, α5(IV) expression was observed between CD31^+^ endothelia and WT1^+^ podocyte-like structures in the 1383D2-derived grafts (Fig. 4b). In contrast, although well-vascularized glomerulus-like structures were observed, no α5(IV) expression was found in mild or severe AS grafts (Fig. 4b). These results further indicate that our kidney organoids recapitulate AS phenotypes in GBMs.

### Chemical chaperone treatment restores α5(IV) conformation in mild AS kidney organoids

**Fig. 5:**
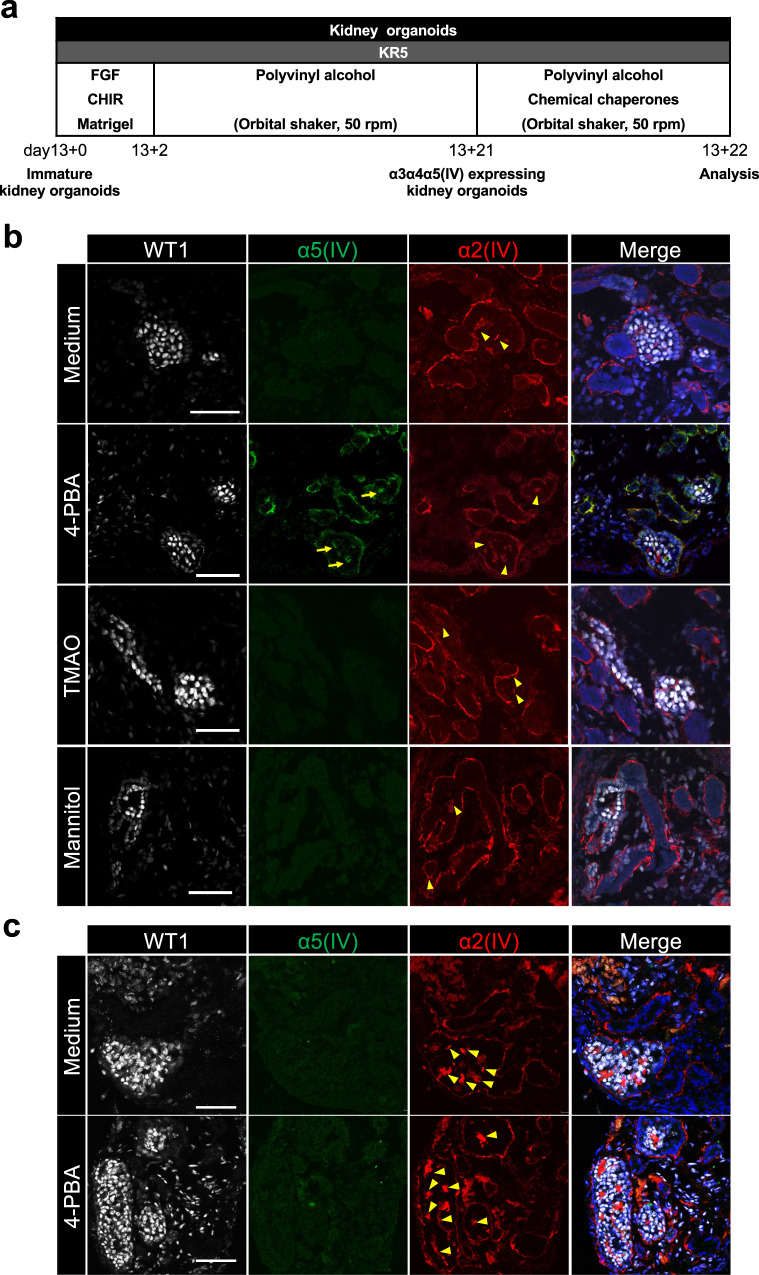
Treatment with 4-PBA restores α5(IV) expression in mild AS patient iPSC-derived kidney organoids. **a** Experimental protocol. **b** Immunostaining images of glomerulus-like structures in mild AS patient iPSC-derived kidney organoids treated with medium, 4-PBA, TMAO, and mannitol for WT1, α5(IV), and α2(IV). **c** Immunostaining images of glomerulus-like structures in severe AS patient iPSC-derived kidney organoids treated with medium and 4-PBA for WT1, α5(IV), and α2(IV). Arrows and arrowheads indicate α5(IV)- and α2(IV)-expressing GBM-like structures, respectively. Scale bars, 50 µm.

While α3α4α5(IV) heterotrimers are assembled in the endoplasmic reticulum (ER) and secreted to the extracellular space via the Golgi apparatus, unassembled or misassembled α3α4α5(IV) heterotrimers cause ER stress and autophagy activation, which leads to their degradation^44,45^. A previous study reported that chemical chaperone treatment in *COL4A2*-mutated patient primary dermal fibroblasts reduces the intracellular accumulation of collagen IV mutants and attenuated ER stress, although the study did not reveal whether chemical chaperone treatment rescues basement membrane integrity^46^. Chemical chaperones potentially normalize misfolded proteins and are candidate AS drugs, accelerating the extracellular secretion of mutated α3α4α5(IV) or ameliorating ER stress by modulating collagen secretion that stacks in the ER^44,45^. Because our results suggested that α3α4α5(IV) heterotrimers with structural abnormalities can form GBM-like structures in mild AS (Fig. 3c), we selected mild AS kidney organoids to examine chemical modifications in the α3α4α5(IV) formation. We treated mild AS kidney organoids with three representative low-molecular-weight chemical chaperones: 4-phenyl butyric acid (4-PBA), trimethylamine N-oxide (TMAO) and mannitol (Fig. 5a) for 1 day, and confirmed α5(IV) expression in the GBM-like structures after 4-PBA exposure (Fig. 5b). However, 4-PBA treatment did not restore α5(IV) expression in severe AS kidney organoids (Fig. 5c). These data suggest that 4-PBA has the potential to correct α5(IV) abnormalities and/or restore α3α4α5(IV) heterotrimer folding. Therefore, because the chaperone effects of collagen trimerization differ depending on the type of *COL4A5* mutation^44,45^, our AS patient iPSC-based kidney organoids are useful for predicting the efficacy of chemical chaperones as personalized medicine.

It was reported that laminin α2-defective basement membrane in skeletal muscle can be rescued by the expression of laminin α1 chain^47^. Similarly, AS symptoms may be attenuated by augmenting GBM strength with other collagen subunits. In AS patients and mouse models, the compensatory expression of α1α1α2(IV) in GBM was reported^48, 49^. Although a compensatory increase in α1(IV) may contribute to increased GBM strength, previous studies using a *Col4a3* and *Ddr* double knockout AS mouse model suggested that the Ddr pathway is crucial for the pathogenesis^50,51^. Since α1(IV) stimulates Ddr, the compensatory expression of α1(IV) may activate Ddr signaling and exacerbate the symptoms. Another study showed that the overexpression of *Col4a3* in vascular endothelial cells does not restore the loss of α3α4α5(IV)^52^. Thus, it remains unclear whether the expression of only a single subunit has therapeutic effects. Epigenetic regulatory treatments may increase the expression of mutated α3α4α5(IV) or α1α1α2(IV). However, no studies have attempted to epigenetically regulate chromatin structures and induce the expression of α3α4α5(IV) or α1α2α2(IV).

In conclusion, we succeeded in creating an in vitro disease model using iPSC-based kidney organoids that recapitulate the characteristics of early-stage AS. Our model reproduces type IV collagen switching in GBMs during embryonic development and patient-specific α5(IV) and α3(IV) defects in GBM-like structures. A low-molecular-weight compound, 4-PBA, improved α5(IV) protein expression in the GBM-like structures of our kidney organoids, indicating that these organoids are potentially applicable to drug screening for restoring GBM abnormalities in AS towards patient-specific personalized medicine. Thus, these findings demonstrate the resourcefulness of patient iPSC-based organoids for the study of AS disease mechanisms and related drug discovery.

## Methods

### Inclusion and ethics

Experiments using iPSCs were approved by the Ethics Committees of Kyoto University, Kobe University, and Taisho Pharmaceutical Co., Ltd. All patients and donors from whom iPSCs were derived provided written informed consent. Animal experiments were approved by the CiRA Animal Experiment Committee and conducted in accordance with institutional guidelines.

### Generation of patient iPSC lines

The iPSC lines were generated from two male XLAS patients based on a previously reported protocol^29,30^. PBMCs were obtained from the patients at the Department of Pediatrics, Kobe University Graduate School of Medicine, and reprogrammed by introducing pCEhOct4, pCEhSK, pCEhUL, pCEmp53DD, and pCXB-EBNA1, which encoded OCT3/4, SOX2, KLF4, L-MYC, LIN28, and mouse p53DD under feeder-free culture conditions.

### Animal experiments

12-20-week-old female G5X AS model mice (B6.Cg-Col4a5^tm1Yseg^/J), 10–30-week-old male C57BL/6 mice, and 8–12-week-old male NOD/SCID mice were maintained at an SPF animal facility in CiRA. E15.5 and E18.5 male embryos from G5X AS model mice were obtained by crossing female G5X AS model mice and male C57BL/6 mice. For genotyping, genomic DNA was extracted from the ears or tails using the KANEKA Easy DNA Extraction Kit version 2 (KANEKA), and sequencing analysis was performed. The primers used are described in Supplementary Table 2. The renal subcapsular transplantation of NPCs was conducted based on our previously established protocol^25^. Induced NPCs on day 9 were seeded in low-binding 96U-plates at 1 × 10^4^ cells/well and cultured for 4 days. Then, approximately 120 NPC aggregates were transplanted into renal subcapsules of NOD/SCID mice under systemic anesthesia. Day 21 grafts were used for the analysis.

### Cell culture and karyotype analysis of iPSCs

All iPSC lines were cultured in StemFit_®_ AK02N (Ajinomoto) and iMatrix-511 silk (Matrixome) under feeder-free conditions^30^. 4 × 10^4^ cells were seeded on a six-well plate (Greiner) and passaged with the EDTA method^53^ every 4 days. The cells were routinely tested for mycoplasma contamination. All iPSC lines generated in this study were examined for their karyotypes by LSI Medience (Tokyo, Japan).

### Sequencing analysis

For Sanger sequencing, genomic DNA was extracted from iPSCs using Cell Lysis Solution and Protein Precipitation Solution in Gentra Puregene Kits (QIAGEN). Extracted crude genomic DNA was precipitated with isopropanol and washed with ethanol for purification. The sequence around exon 24 of *COL4A5* was amplified with the standard polymerase chain reaction (PCR) protocol using the primer sequences listed in Supplementary Table 2. After purification of the PCR products using the MinElute Reaction Cleanup Kit (QIAGEN), sequencing was performed using BigDye™ and analyzed using an Applied Biosystems^TM^ 3500/3500xL Genetic Analyzer.

### iPSC differentiation into NPCs and kidney organoids

We slightly modified our previously published protocol for NPC induction from iPSCs^25^. iPSCs cultured in StemFit_®_ AK02N and iMatrix-511 silk were differentiated into NPCs using DMEM/F12 GlutaMAX^TM^ (Thermo Fisher Scientific) supplemented with B27 supplement minus vitamin A (Thermo Fisher Scientific) on a 24-well plate (Greiner) from days 0 to 9 and on a low-binding 96-well U plate (Sumitomo Bakelite) from days 9 to 13. Then, the basal culture medium was switched to KR5 medium, which is DMEM/F12 GlutaMAX^TM^ containing 0.1 mM non-essential amino acids (Thermo Fisher Scientific), 55 µM 2-mercaptoethanol (Thermo Fisher Scientific), and 5% Knockout serum replacement (Thermo Fisher Scientific). NPC aggregates on day 13 were incubated for 2 days in a 96-well U plate using KR5 medium containing 200 ng/mL fibroblast growth factor (FGF)9 (Peprotech), 1 µM CHIR99021 (Axon) and 2% Matrigel (BD) for the organoid formation. Further organoid maturation steps were performed in KR5 medium containing 0.05% polyvinyl alcohol (Sigma-Aldrich) in a low-binding six-well plate (Iwaki) using an orbital shaker (CS-LR; Taitec).

### Chemical chaperone treatment

Low-molecular-weight chemical chaperone compounds were purchased from the suppliers listed in Supplementary Table 1. Kidney organoids were treated for 24 hours with 4-PBA (10 mM), TMAO (150 mM), or mannitol (150 mM), which were dissolved in KR5 medium and filtrated before use.

### Immunofluorescence analysis

Immunofluorescence analysis was conducted based on our previously established protocol^25^. Cells were fixed with 4% PFA/phosphate buffer (PB) for 15 min at 4 °C. Kidney organoids were fixed with 4% PFA/PB for 30 min at 4 °C or ice-cold acetone for 15 min, embedded into OCT compounds (Sakura Finetek Japan), and frozen quickly at −80 °C. Then, 4-µm frozen sections were prepared using a CM1520 cryostat (Leica). Primary antibodies were diluted in 5% donkey serum/PBST (PBS/0.3% Triton-X100) and incubated overnight at 4 °C. After washing with PBST, secondary antibodies were incubated for 1 hour at room temperature (RT). Stained samples were observed using a confocal microscope, FV-3000 (Olympus). The immunofluorescence reagents used in this study are listed in Supplementary Table 1.

### Induction rates

We adopted a quantitative immunofluorescence analysis to verify the NPC induction rate. NPCs induced on day 9 or 13 were seeded in 96-well plates at 3–5 × 10^4^ cells/well and incubated for three hours. Then, fixed NPCs were stained by anti-WT1 or anti-PAX2 antibodies listed in Supplementary Table 1. Immunofluorescence data were obtained using a BZ-X700 and analyzed by a BZ-X Analyzer (KEYENCE).

### Preparation of CRISPR sgRNA by in vitro transcription (IVT)

We designed CRISPR-Cas9 gRNA for the c.1634 G > A (p.G545D) missense mutation on the *COL4A5* gene and performed ssODN-mediated homologous recombination based on a previously published protocol^54,55^. The PCR template for IVT reaction to prepare gRNA was prepared using the following two primers: forward, 5′-GAAATTAATACGACTCACTATAGGCCTGGATCTAAAGATGAACCGTTTTAGAGCTAGAAATAGCAAG-3′; and reverse, 5′-AAAGCACCGACTCGGTGCCACTTTTTCAAGTTGATAACGGACTAGCCTTATTTTAACTTGCTATTTCTAGCTCTAAAAC-3′. The purified PCR product was used for the IVT reaction using the MEGAshortscript™ T7 Transcription Kit (Thermo Fisher Scientific) for 6 hours at 37 °C. Then, 2 µL of TURBO^TM^ DNase (Thermo Fisher Scientific) was added and incubated for 30 min at 37 °C to remove the DNA template. The sgRNA produced by the IVT reaction was purified using the QIAGEN RNeasy MinElute Cleanup Kit (QIAGEN).

### CRISPR-Cas9- and ssODN-mediated correction of *COL4A5* mutation in iPSCs

For electroporation, semi-confluent iPSCs (mild AS patient) cultured with StemFit AK02N media in iMatrix-511-coated six-well plates were washed with 2 mL PBS and incubated with 0.5 mL TrypLE Select for 10 min at 37 °C. The cells were then detached from the plate by pipetting and transferred to a 1.5 mL tube containing StemFit AK02N media with 10 µM Y-27632 (Wako). After counting the number of cells, 3 × 10^5^ cells were transferred to a 1.5 mL tube per sample and centrifuged for 5 min at 120 × *g*. The cell pellet was resuspended in 20 µL P4 Primary Cell Nucleofector Solution from a P4 Primary Cell 4D-Nucleofector Kit (Lonza). For RNP electroporation, 5 µg of recombinant SpCas9 protein (Thermo Fisher Scientific) and 1.25 µg of sgRNA prepared by the IVT reaction (as described above) were incubated for 5 min at RT and then added to the cell suspension with 6 µg of ssODN (5′-TCAGGGCATTCCAGGAGCTCCAGGTGCTCCAGGCTTTCCTGGATCTAAAGgTGAACCTGGTGATATCCTCACTTTTCCAGGAATGAAGGGTGACAAAGGA-3′). The cell suspension was transferred to a Nucleocuvette Strip and electroporated using the CA-137 protocol of 4D-Nucleofector. Then, the cells were cultured in iMatrix-511-coated six-well plates containing 2 mL of AK02N with 10 µM Y-27632 per well. Three to 7 days post-transfection, some cells were expanded for further culture, and the other cells were harvested for genomic DNA extraction. The target region on the *COL4A5* gene was PCR amplified from the genomic DNA using the forward primer 5′-CATGCCTCACTTGATTCAGCC-3′ and reverse primer 5′-CAGCATCAGTCCCATCCTTTG-3′ for Sanger sequencing. After confirmation of the desired genetic correction of the bulk cells by Sanger sequencing, we isolated subclones by limiting dilution and established several corrected subclones confirmed by Sanger sequencing.

### Transmission electron microscopy

After washing with PBS, day 13 + 28 kidney organoids and mouse embryonic kidneys were fixed with 1.4% PFA/1% glutaraldehyde/0.1 M PB overnight at 4 °C. Then, the organoids were washed with isotonic phosphate-buffered sucrose, refixed with phosphate-buffered 1% OsO_4_, dehydrated through a graded series of ethanol solutions, and embedded in Luveak 812 (Nacalai Tesque). Thin sections (70–90 nm thick) were cut with a diamond knife on an EM UC7 ultramicrotome (Leica), stained with uranyl acetate and lead citrate, and observed using a JEM-1400Flash electron microscope (JEOL).

### Real-time quantitative RT-PCR (qRT-PCR)

Kidney organoids were harvested and lysed in buffer RLT using BioMasher II (Nippi). Total RNA was extracted using an RNeasy Kit (QIAGEN) according to the manufacturer’s protocol. cDNA was synthesized using ReverTra Ace (TOYOBO), and qPCR was performed with SYBR Green PCR Master Mix (Takara) using QuantStudio 3 (Thermo Fisher Scientific). The PCR reactions were performed in triplicate for each sample. The primer sequences used in this study are listed in Supplementary Table 2.

### RNA sequencing

For RNA sequencing, total RNA was extracted as described above. The samples preserved at −80 °C were shipped and analyzed by DNAFORM. The quality of total RNA was evaluated by a Bioanalyzer (Agilent) to ensure over 8.0 RIN (RNA integrity number) or by electrophoresis waveforms. Double-stranded cDNA libraries (RNA-seq libraries) were prepared using a SMART Seq Stranded Kit (Clontech) according to the manufacturer’s protocols. RNA-seq libraries were sequenced using paired-end reads (50 nt of read 1 and 25 nt of read 2) on a NextSeq 500 (Illumina). Obtained reads were mapped to the human GRCh38 genome analyzed by STAR (version 2.7.3a). Annotated reads were counted using featureCounts (version 2.0.1) and RSEM (version 1.3.1). FPKM values were calculated from mapped reads by normalizing to total counts. The gene expression heatmap was drawn based on the log2 fold change (log2FC) compared with day 13 + 4 kidney organoid samples using FPKM.

### Flow cytometry

Kidney organoids were dissociated in TrypLE Select Enzyme (Thermo Fisher Scientific) for approximately 30 min at 37 °C. The cell suspension was incubated with primary antibodies in 2% fetal bovine serum (FBS; Wako)/PBS for 30 min on ice. After washing, the cell suspension was incubated with secondary antibodies for 30 min on ice. Antibody-labeled cells were resuspended with 2% FBS/PBS containing 4’,6-diamidino-2-phenylindole (DAPI; Sigma). Flow cytometry was performed using a BD FACSAria II (BD Biosciences). The FACS Diva (BD) software program was used to analyze the data. The cells stained with isotype control and secondary antibodies were used as a negative control. Gating was set such that DAPI(−) live negative control cells had a positive fraction of less than 1%.

### Statistics and reproducibility

All quantitative data are represented as the mean ± SE. Student’s *t* test was used to compare the means of two groups, and Dunnett’s test or Tukey–Kramer test was used for multiple comparisons among groups. *p* < 0.05 was considered statistically significant for all analyses. *p < 0.05, **p < 0.01, and ***p < 0.001 in the figures.

### Reporting summary

Further information on research design is available in the Nature Portfolio Reporting Summary linked to this article.

### Supplementary information

Supplementary Information

Description of Additional Supplementary Files

Supplementary Data 1

Reporting Summary

## Data Availability

The NCBI GEO accession number for the RNA sequencing data in this paper is GSE236314. All other data are available from the corresponding author upon reasonable request. The source data for the graphs are available in Supplementary Data 1.
