# Supplementary material for: iPSC-derived type IV collagen α5-expressing kidney organoids model Alport syndrome
Source: Commun Biol. 2023 Sep 28;6:854. doi: 10.1038/s42003-023-05203-4 (PMC10539496; doi:10.1038/s42003-023-05203-4)
Supplement: Supplementary file 1 — Supplementary Information [file 42003_2023_5203_MOESM1_ESM.pdf]

## Supplementary information

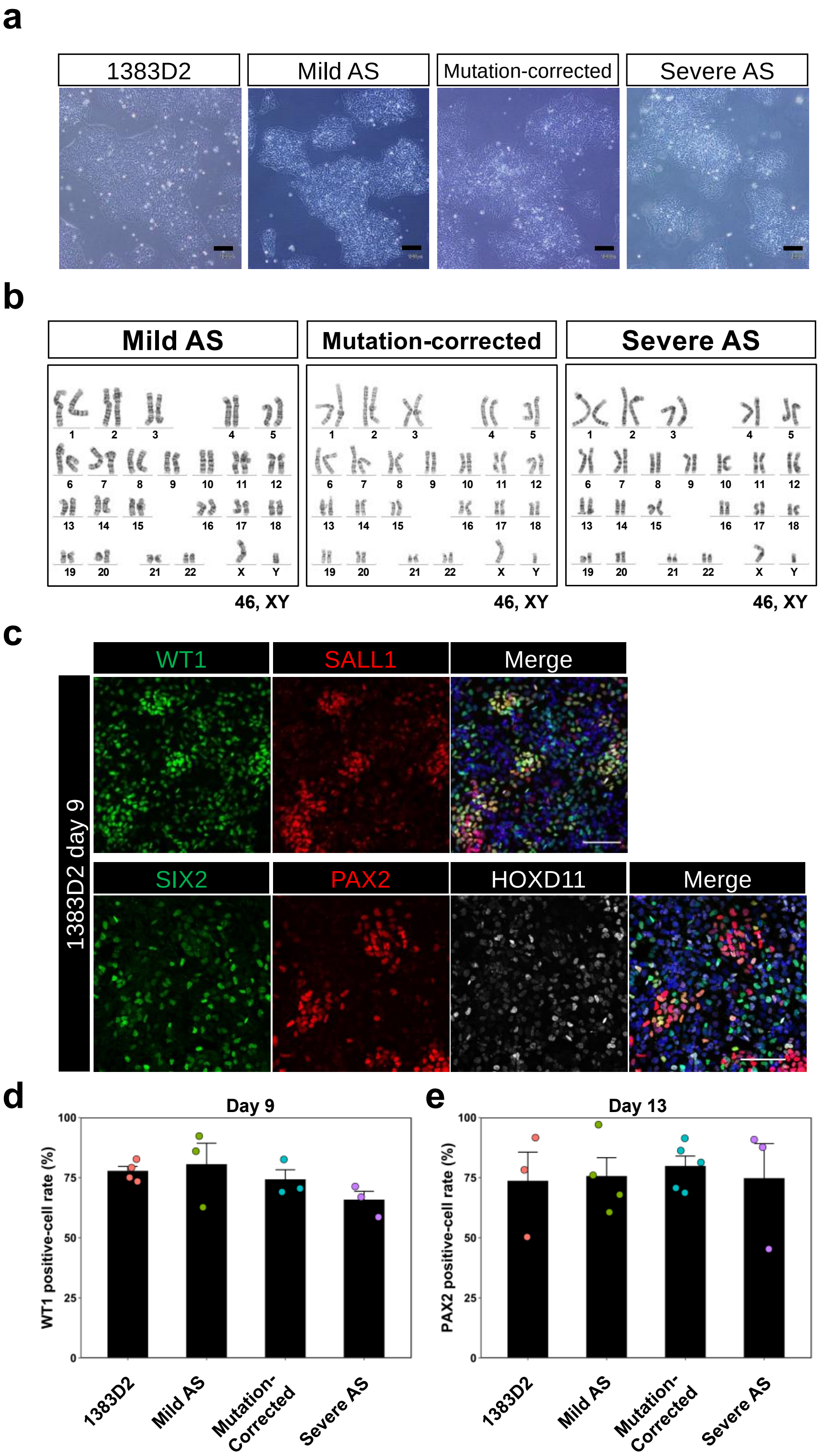


**Figure S1. Morphology and karyotype of AS patient iPSCs and differentiation into NPCs.** related to Figure 1.

(a) The morphology of iPSC colonies under bright field microscopy. Scale bars, 100 µm. (b) Mild AS patient iPSCs, mutation-corrected mild AS patient iPSCs and severe AS patient iPSCs show normal karyotype of 46, XY. (c) Immunostaining images of 1383D2-derived day 9 differentiated cells for the NPC markers WT1, SALL1, SIX2, PAX2 and HOXD11. Blue

indicates nuclei stained by Hoechst 33342. Scale bars, 100 μm. (d, e) Induction rate of WT1$^{+}$ cells on day 9 (d) and PAX2$^{+}$ cells on day 13 (e) from 1383D2 cells, mild AS patient iPSCs, mutation-corrected mild AS patient iPSCs and severe AS patient iPSCs. The data from three to five independent experiments are represented as the means ± SEM in (d) and (e).

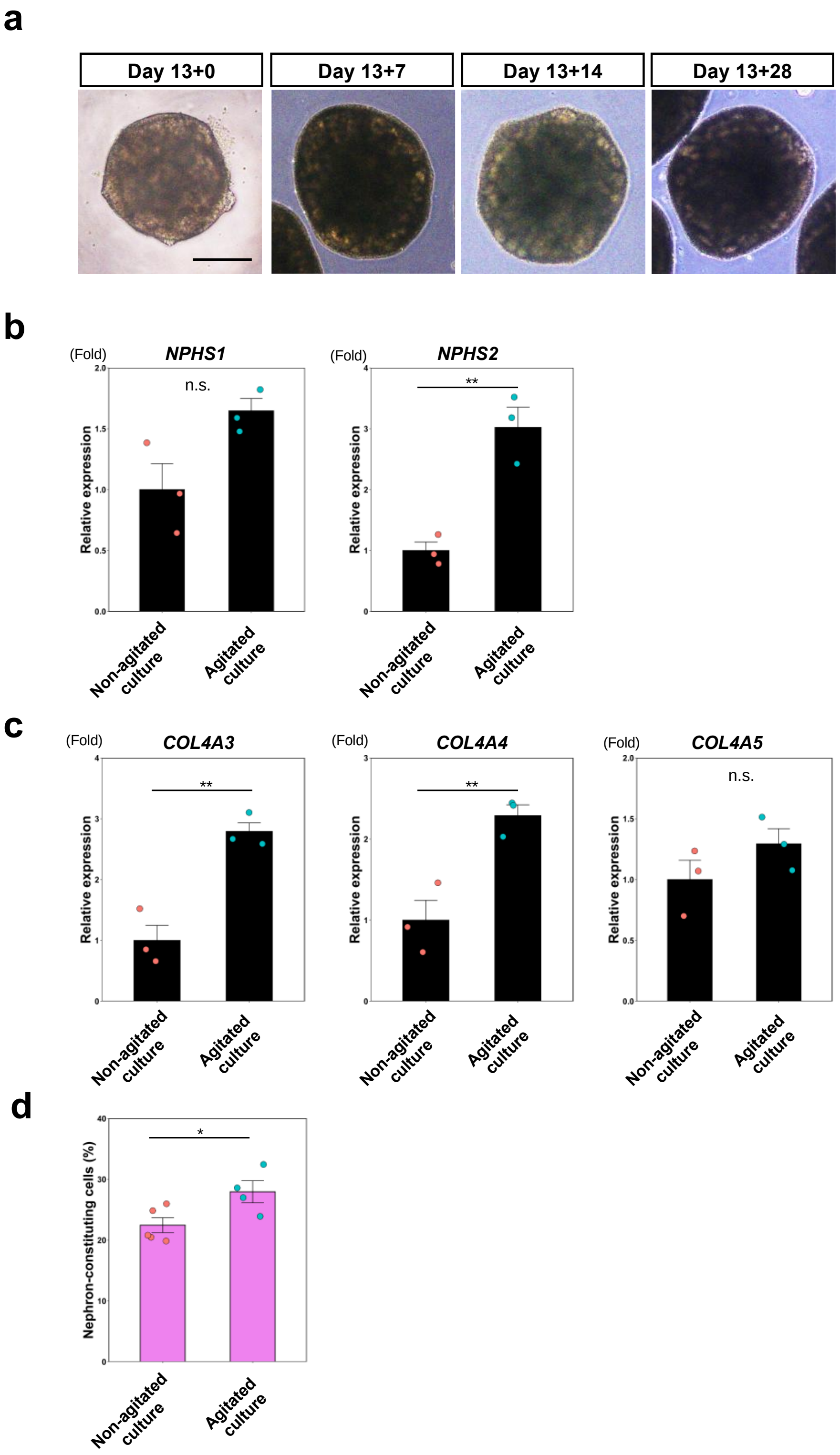


**Figure S2. Agitated culture with an orbital shaker promotes organoid formation.** related to Figure 1.

(a) Bright field images of 1383D2-derived kidney organoids on days 13+0 to 13+28. Scale bar, 300 μm. (b) qRT-PCR analysis for the expression of the podocyte markers *NPHS1* and *NPHS2* in 1383D2-derived day 13+28 kidney organoids generated with or without agitated cultures using an orbital shaker. (c) qRT-PCR analysis for the expression of *COL4A3*, *COL4A4* and *COL4A*5 in 1383D2-derived day 13+21 kidney organoids generated with or without agitated

cultures. (d) Flow cytometry analysis of 1383D2-derived day 13+28 kidney organoids generated with (n=4) or without agitated cultures (n=5) for nephron-constituting cells (total $PODXL^+$ or $EpCAM^+$ populations). The data from three independent experiments are represented as the means ± SEM in (b) - (d). (n=3) in (b) and (c). *$p<0.05$, **$p<0.01$, by Student’s *t*-test, n.s., not statistically significant.

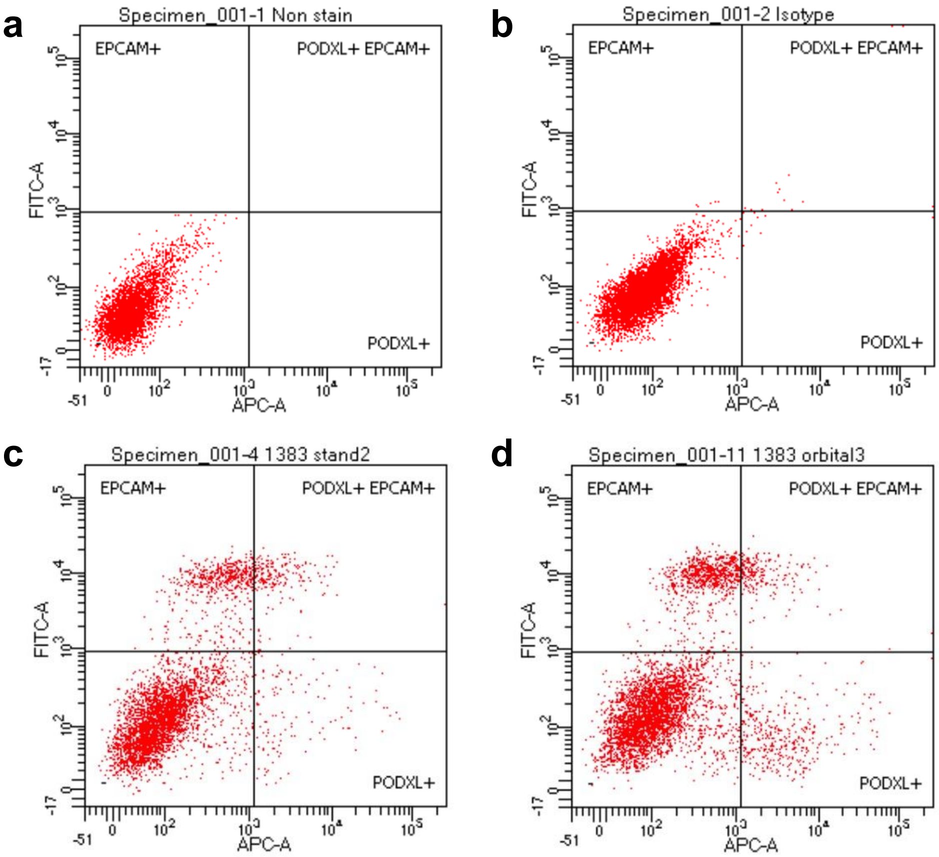


**Figure S3. Flow cytometry gating strategy.** related to Figure S2d.

(a, b) Representative flow cytometry plots of 1383D2-derived day 13+28 kidney organoids without antibody staining (a) or stained with isotype control antibodies (b). (c, d) Representative flow cytometry plots of 1383D2-derived day 13+28 kidney organoids generated without (c) or with agitated cultures (d). The organoids were stained with anti-PODXL and anti-EpCAM antibodies.

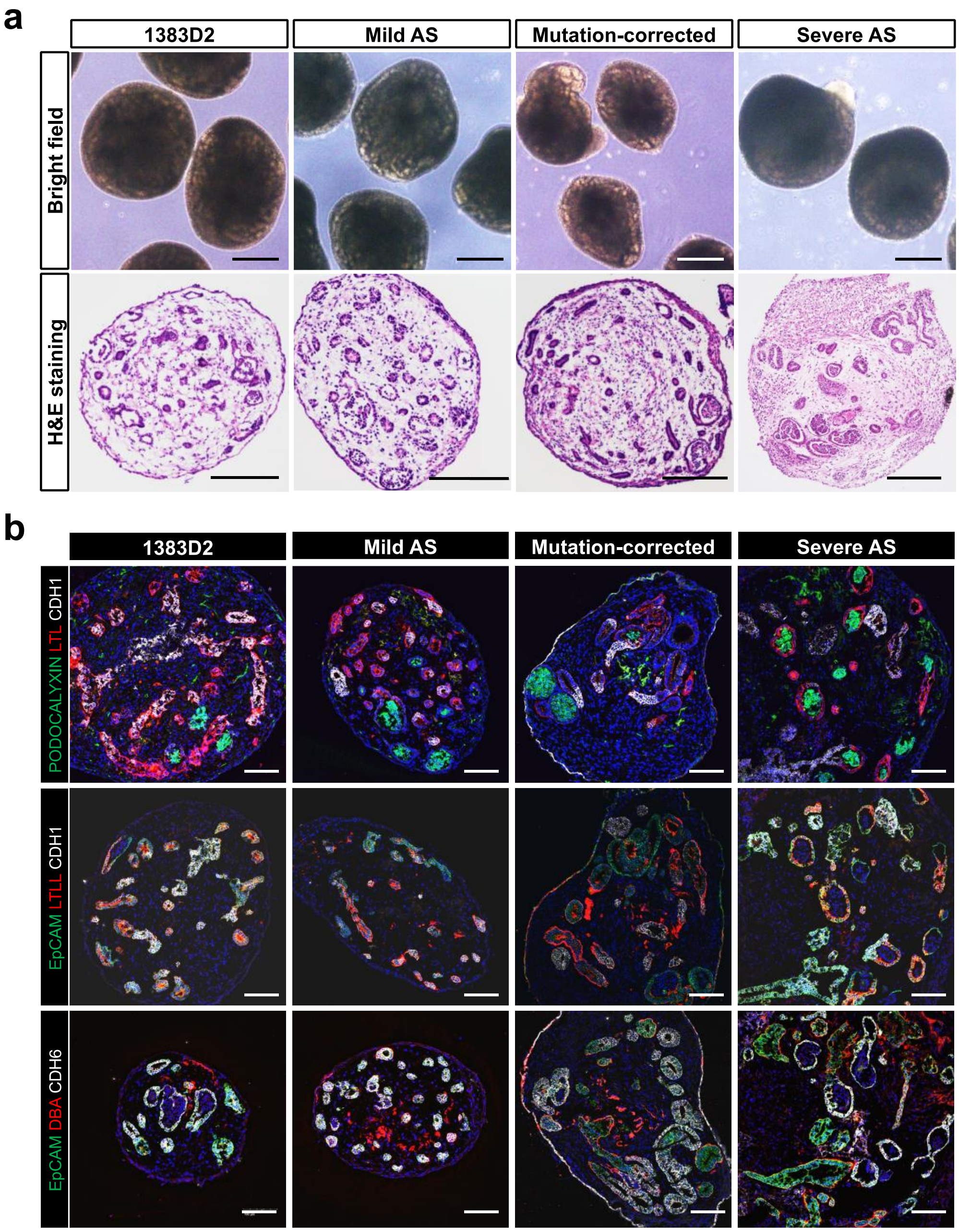


**Figure S4. Comparison of kidney organoids derived from healthy and AS patient iPSCs.** related to Figure 1.

(a) Bright field images of day 13+21 kidney organoids and low magnification H&E staining images of day 13+28 kidney organoids differentiated from 1383D2 cells, mild AS patient iPSCs, mutation-corrected mild AS patient iPSCs and severe AS patient iPSCs. Scale bars, 300 µm in bright field images and 200 µm in H&E staining images. (b) Immunostaining images of day 13+28 kidney organoids from 1383D2 cells, mild AS patient iPSCs, mutation-corrected mild AS patient iPSCs and severe AS patient iPSCs for PODOCALYXIN, LTL and CDH1, for EpCAM (tubular epithelial cells), LTL and CDH1, and for EpCAM, DBA and CDH6 (proximal tubules). Blue indicates nuclei stained by Hoechst 33342. Scale bars, 100 µm.

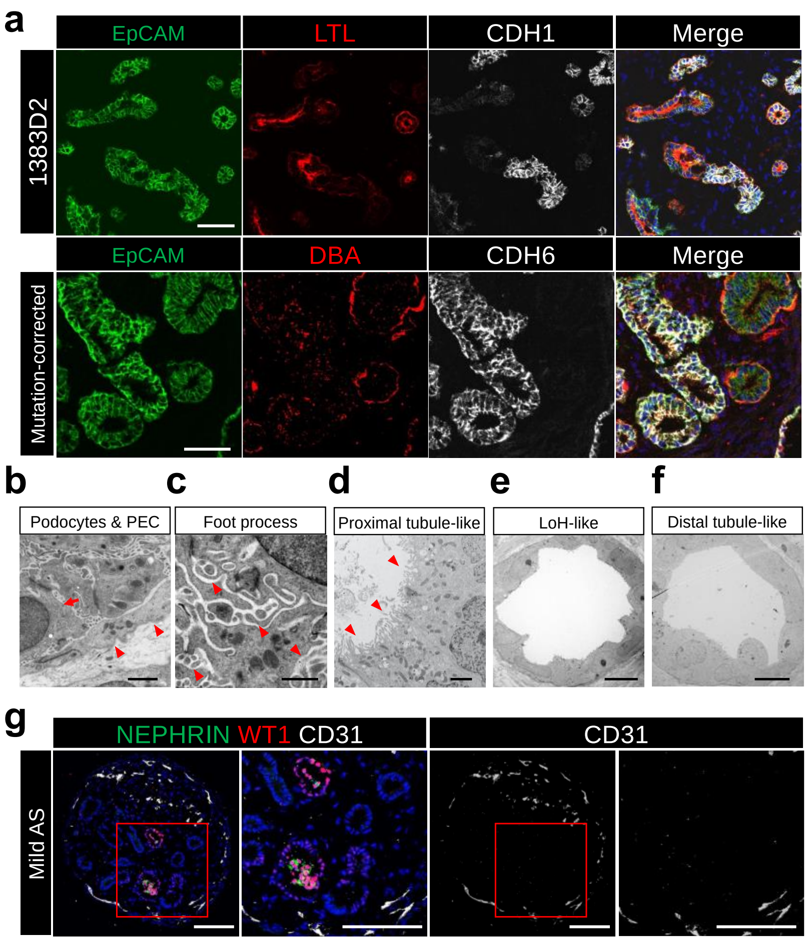


**Figure S5. Characterization of kidney organoids.** related to Figure 1.

(a) Immunostaining images of a 1383D2-derived day 13+28 kidney organoid for EpCAM, LTL and CDH1 and of a mutation-corrected mild AS patient iPSC-derived day 13+28 kidney organoid for EpCAM, DBA and CDH6. Blue indicates nuclei stained by Hoechst 33342. Scale bars, 50 µm. (b) - (f) TEM analysis of day 13+28 kidney organoids from 1383D2 cells in (b) - (d) and (f) and from mild AS patient iPSCs in (e) for podocyte-like cells (arrow) and parietal epithelial cell (PEC)-like cells (arrowheads; b), foot processes (arrowheads) of podocyte-like cells (c), proximal tubule-like structures with brush borders (arrowheads; d), loop of Henle-like structures (e) and distal tubule-like structures (f). Scale bars, 2 µm in (b) and (d), 1 µm in (c), and 10 µm in (e) and (f). (g) Immunostaining images of a mild AS patient iPSC-derived day 13+28 kidney organoid for NEPHRIN, WT1 (podocytes) and CD31 (endothelial cells). Magnified images of the red boxed areas are also shown. Blue indicates nuclei stained by Hoechst 33342. Scale bars, 100 µm in low magnification images and 50 µm in magnified images.

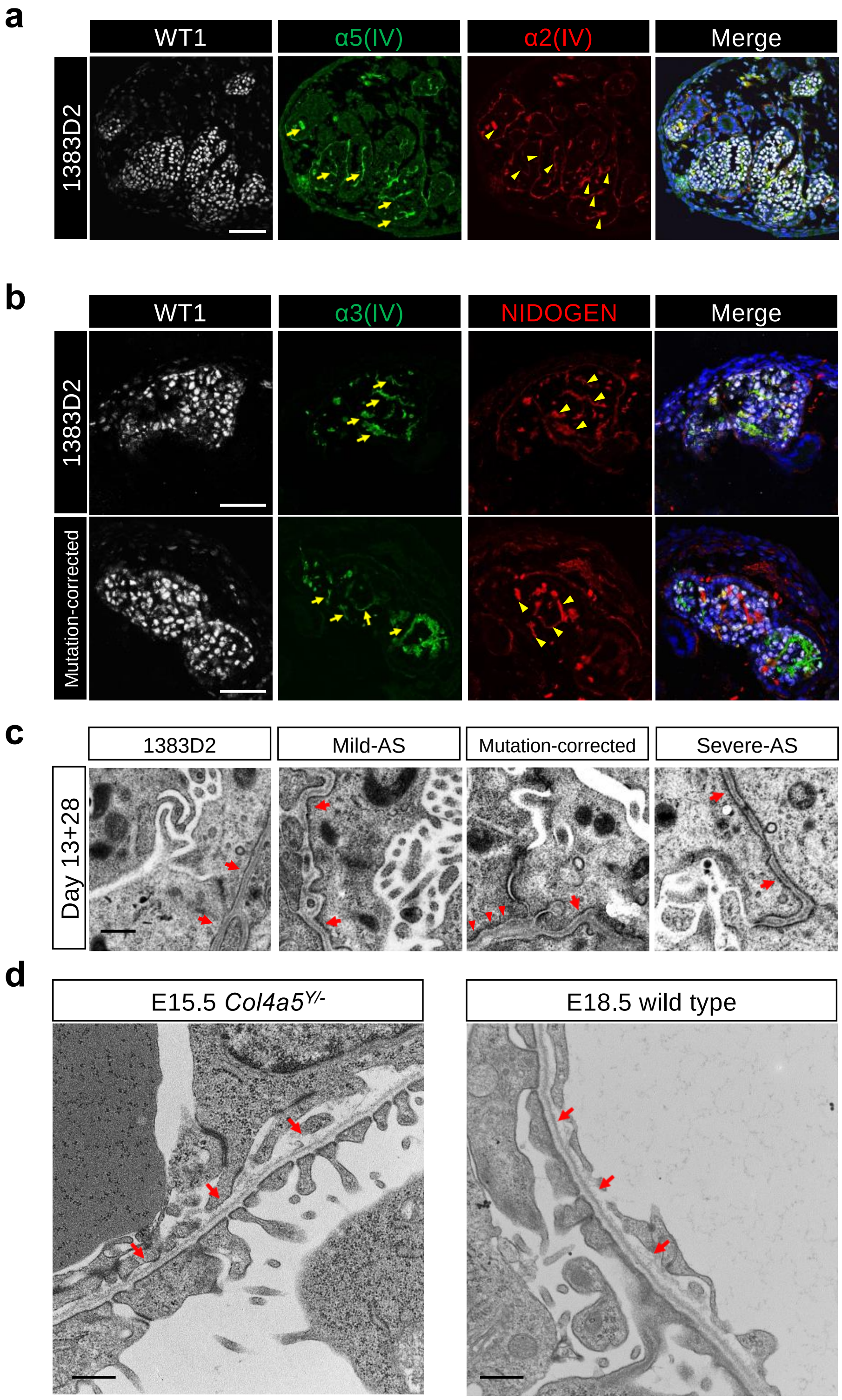


**Figure S6. Collagen composition of GBM-like structures in kidney organoids and comparison of AS patient iPSC-derived kidney organoids and embryonic kidneys of AS model mice.** related to Figures 2 and 3.

(a) Low magnification immunostaining images of a 1383D2-derived day 13+28 kidney organoid for WT1, α5(IV) and α2(IV). Arrows and arrowheads indicate α5(IV)- and α2(IV)-expressing GBM-like structures, respectively. Blue indicates nuclei stained by Hoechst 33342. Scale bar, 50 µm. (b) Immunostaining images of glomerulus-like structures in day 13+28

kidney organoids from 1383D2 cells and mutation-corrected mild AS patient iPSCs for WT1, α3(IV) and NIDOGEN. Arrows and arrowheads indicate α3(IV)- and NIDOGEN-expressing GBM-like structures, respectively. Blue indicates nuclei stained by Hoechst 33342. Scale bars, 50 μm. (c, d) TEM analysis of GBM-like structures (arrows) and split GBM-like structures (arrowheads) in day 13+28 kidney organoids from 1383D2 cells, mild AS patient iPSCs, mutation-corrected mild AS patient iPSCs and severe AS patient iPSCs (c) and GBMs (arrows) in embryonic kidneys from E15.5 *Col4a5*$^{Y/-}$ and E18.5 wild-type mice (d). Scale bars, 500 nm in (c) and (d).

**Supplementary table 1—key resources table**

| REAGENT or RESOURCE | SOURCE | IDENTIFIER |
|---|---|---|
| **Antibodies** | | |
| Mouse anti-E-CADHERIN | BD | Cat#610181 |
| Mouse anti-CADHERIN6 | R&D | Cat#MAB2715 |
| Goat anti-PAX2 | R&D | Cat#AF3364 |
| Anti collagen IV cocktail for Alport's syndrome | Shigei Med.Res.Inst. | Cat#CFT-45325 |
| Anti collagen IV α5(IV) clone B51 | Shigei Med.Res.Inst. | Cat#SGE-C-451 |
| Collagen IV α5(IV) clone H53 | Shigei Med.Res.Inst. | Cat#SGE-C-453 |
| Rat anti-α3(IV) clone H31 | Chondrex | Cat#7076 |
| Rabbit anti-WT1 | Abcam | Cat#ab89901 |
| Goat anti-CD31 | R&D | Cat#AF3628 |
| Goat anti-NANOG | R&D | Cat#AF1997 |
| Guinea pig anti-NEPHRIN | Progen | Cat#POG-GP-N2 |
| Rat anti-LAMININ β1 | Invitrogen | Cat#MA5-14657 |
| Mouse anti-NIDOGEN | Santa Cruz Biotechnology | Cat#sc-133175 |
| Goat anti-PODOCALYXIN | R&D | Cat#AF1658 |
| LTL-biotinylated | Vector lab | Cat#B1325 |
| DBA-biotinylated | Vector lab | Cat# B-1035 |
| Mouse anti-EpCAM | CST | Cat#2929S |
| Rabbit anti-EpCAM | CST | Cat#36746S |
| Goat anti-SIX2 | Proteintech | Cat#11562-1-AP |
| Mouse anti-SALL1 | Perseus Proteomics | Cat#PP-K9814-00 |
| Mouse IgG Isotype Control | Invitrogen | Cat#31903 |
| Normal Goat IgG Control | R&D | Cat#AB-108-C |
| Donkey anti-Mouse IgG- Alexa Fluor 488 | Thermo Fisher Scientific | Cat#A21202 |
| Donkey anti-Rabbit IgG- Alexa Fluor 488 | Thermo Fisher Scientific | Cat#A21206 |
| Donkey anti-Goat IgG- Alexa Fluor 488 | Thermo Fisher Scientific | Cat#11055 |
| Donkey anti-Guinea pig IgG- Alexa Fluor 488 | Jackson ImmunoResearch | Cat#706-545-148 |
| Donkey anti-Mouse IgG- Alexa Fluor 546 | Thermo Fisher Scientific | Cat#A10036 |

| | | |
|---|---|---|
| Donkey anti-Rabbit IgG- Alexa Fluor 546 | Thermo Fisher Scientific | Cat#A10040 |
| Donkey anti-Goat IgG- Alexa Fluor 546 | Thermo Fisher Scientific | Cat#A11056 |
| streptavidin, Alexa Fluor 546 conjugate | Thermo Fisher Scientific | Cat#S11225 |
| Donkey anti-Mouse IgG- Alexa Fluor 647 | Thermo Fisher Scientific | Cat#A31571 |
| Donkey anti-Rabbit IgG- Alexa Fluor 647 | Thermo Fisher Scientific | Cat#A31573 |
| Donkey anti-Goat IgG- Alexa Fluor 647 | Thermo Fisher Scientific | Cat#A21447 |
| Hoechst 33342 | Thermo Fisher Scientific | Cat#H1399 |
| **Reagents** | | |
| 4-Phenylbutyric acid (4-PBA) | Sigma | Cat#P21005 |
| Trimethylamine N-Oxide (TMAO) | Sigma | Cat#317594 |
| Mannitol | Nacalai tesque | Cat#11662-42 |
| **Cell lines** | | |
| Human: 1383D2 iPSC line | ICSCB | HPS1005 |
| Mild AS patient iPSC line | CiRA, Kyoto University | CiRA00878-2 |
| Mutation-corrected mild AS patient iPSC line | CiRA, Kyoto University | CiRA00878-2-re15 |
| Severe AS patient iPSC line | CiRA, Kyoto University | CiRA01058-2 |

**Supplementary table 2—Primers used for qRT-PCR and DNA sequencing in this study.**

| Primer | Sequence | |
|---|---|---|
| *Col4a5* for genotyping | ACCACACTGACCACAGTTCAA | CCACCCAGGGTAGCTGTCTA |
| *COL4A5* for sequencing | CATGCCTCACTTGATTCAGCC | CAGCATCAGTCCCATCCTTTG |
| *COL4A5* for qPCR | GCCCCCTGTTTTAATGTTTC | GTACAAGCAGAGGCCACAG |
| *GAPDH* | TGGAAATCCCATCACCATC | TGGACTCCACGACGTACTC |
| *NPHS1* | CTGAGGTCAGGAGTTCGAG | CGCCACTACTCCTGGCTA |
| *NPHS2* | IDT; Hs.PT.58.2682240 | |
| *COL4A3* | IDT; Hs.PT.58.249834 | |
| *COL4A4* | IDT; Hs.PT.58.3212616 | |
