# Supplementary material for: iPSC-derived type IV collagen α5-expressing kidney organoids model Alport syndrome
Source: Commun Biol. 2023 Sep 28;6:854. doi: 10.1038/s42003-023-05203-4 (PMC10539496; doi:10.1038/s42003-023-05203-4)
Supplement: Supplementary file 2 — Description of Additional Supplementary Files [file 42003_2023_5203_MOESM2_ESM.pdf]

Description of Additional Supplementary Files

File name: Supplementary Data 1

Description: The numerical source data for graphs
