# Supplementary material for: iPSC-derived type IV collagen α5-expressing kidney organoids model Alport syndrome
Source: Commun Biol. 2023 Sep 28;6:854. doi: 10.1038/s42003-023-05203-4 (PMC10539496; doi:10.1038/s42003-023-05203-4)
Supplement: Supplementary file 4 — Reporting Summary [file 42003_2023_5203_MOESM4_ESM.pdf]

Corresponding author(s): Kenji Osafune
Last updated by author(s): Jul 31, 2023

Reporting Summary

Nature Portfolio wishes to improve the reproducibility of the work that we publish. This form provides structure for consistency and transparency in reporting. For further information on Nature Portfolio policies, see our Editorial Policies and the Editorial Policy Checklist.

Statistics

For all statistical analyses, confirm that the following items are present in the figure legend, table legend, main text, or Methods section.

n/a	Confirmed	
☐	☒	The exact sample size (*n*) for each experimental group/condition, given as a discrete number and unit of measurement
☐	☒	A statement on whether measurements were taken from distinct samples or whether the same sample was measured repeatedly
☐	☒	The statistical test(s) used AND whether they are one- or two-sided<br>*Only common tests should be described solely by name; describe more complex techniques in the Methods section.*
☒	☐	A description of all covariates tested
☐	☒	A description of any assumptions or corrections, such as tests of normality and adjustment for multiple comparisons
☐	☒	A full description of the statistical parameters including central tendency (e.g. means) or other basic estimates (e.g. regression coefficient) AND variation (e.g. standard deviation) or associated estimates of uncertainty (e.g. confidence intervals)
☐	☒	For null hypothesis testing, the test statistic (e.g. *F*, *t*, *r*) with confidence intervals, effect sizes, degrees of freedom and *P* value noted<br>*Give P values as exact values whenever suitable.*
☒	☐	For Bayesian analysis, information on the choice of priors and Markov chain Monte Carlo settings
☒	☐	For hierarchical and complex designs, identification of the appropriate level for tests and full reporting of outcomes
☒	☐	Estimates of effect sizes (e.g. Cohen's *d*, Pearson's *r*), indicating how they were calculated

Our web collection on statistics for biologists contains articles on many of the points above.

Software and code

Policy information about availability of computer code

Data collection	No software was used.
Data analysis	No software was used.

For manuscripts utilizing custom algorithms or software that are central to the research but not yet described in published literature, software must be made available to editors and reviewers. We strongly encourage code deposition in a community repository (e.g. GitHub). See the Nature Portfolio guidelines for submitting code & software for further information.

Data

Policy information about availability of data

All manuscripts must include a data availability statement. This statement should provide the following information, where applicable:

- Accession codes, unique identifiers, or web links for publicly available datasets
- A description of any restrictions on data availability
- For clinical datasets or third party data, please ensure that the statement adheres to our policy

The NCBI GEO accession number for the RNA sequencing data in this paper is GSE236314. Any further requests can be directed to the corresponding author.

Human research participants

Policy information about studies involving human research participants and Sex and Gender in Research.

Reporting on sex and gender	iPS cells were generated in this study from two male patients with Alport syndrome. Both patients are around 20 years old.
Population characteristics	iPS cells were generated in this study from two male patients with Alport syndrome. Both patients are around 20 years old.
Recruitment	*Describe how participants were recruited. Outline any potential self-selection bias or other biases that may be present and how these are likely to impact results.*
Ethics oversight	Experiments using iPSCs were approved by the Ethics Committees of Kyoto University, Kobe University and Taisho Pharmaceutical Co., Ltd.

Note that full information on the approval of the study protocol must also be provided in the manuscript.

Field-specific reporting

Please select the one below that is the best fit for your research. If you are not sure, read the appropriate sections before making your selection.

☒ Life sciences ☐ Behavioural & social sciences ☐ Ecological, evolutionary & environmental sciences

For a reference copy of the document with all sections, see nature.com/documents/nr-reporting-summary-flat.pdf

Life sciences study design

All studies must disclose on these points even when the disclosure is negative.

Sample size	*Describe how sample size was determined, detailing any statistical methods used to predetermine sample size OR if no sample-size calculation was performed, describe how sample sizes were chosen and provide a rationale for why these sample sizes are sufficient.*
Data exclusions	*Describe any data exclusions. If no data were excluded from the analyses, state so OR if data were excluded, describe the exclusions and the rationale behind them, indicating whether exclusion criteria were pre-established.*
Replication	*Describe the measures taken to verify the reproducibility of the experimental findings. If all attempts at replication were successful, confirm this OR if there are any findings that were not replicated or cannot be reproduced, note this and describe why.*
Randomization	*Describe how samples/organisms/participants were allocated into experimental groups. If allocation was not random, describe how covariates were controlled OR if this is not relevant to your study, explain why.*
Blinding	*Describe whether the investigators were blinded to group allocation during data collection and/or analysis. If blinding was not possible, describe why OR explain why blinding was not relevant to your study.*

Reporting for specific materials, systems and methods

We require information from authors about some types of materials, experimental systems and methods used in many studies. Here, indicate whether each material, system or method listed is relevant to your study. If you are not sure if a list item applies to your research, read the appropriate section before selecting a response.

Materials & experimental systems

n/a	Involved in the study	
☐	☒	Antibodies
☐	☒	Eukaryotic cell lines
☒	☐	Palaeontology and archaeology
☐	☒	Animals and other organisms
☒	☐	Clinical data
☒	☐	Dual use research of concern

Methods

n/a	Involved in the study	
☒	☐	ChIP-seq
☐	☒	Flow cytometry
☒	☐	MRI-based neuroimaging

Antibodies

Antibodies used	Mouse anti-E-CADHERIN, BD, Cat#610181; Mouse anti-CADHERIN6, R&D, Cat#MAB2715; Goat anti-PAX2, R&D, Cat#AF3364; Anti collagen IV cocktail for Alport's syndrome, Shigei Med.Res.Inst., Cat#CFT-45325; Anti collagen IV α5(IV) clone B51, Shigei

Med.Res.Inst., Cat#SGE-C-451; Collagen IV α5(IV) clone H53, Shigei Med.Res.Inst., Cat#SGE-C-453; Rat anti-α3(IV) clone H31, Chondrex, Cat#7076; Rabbit anti-WT1, Abcam, Cat#ab89901; Goat anti-CD31, R&D, Cat#AF3628; Goat anti-NANOG, R&D, Cat#AF1997; Guinea pig anti-NEPHRIN, Progen, Cat#POG-GP-N2-100; Rat anti-LAMININ β1, Invitrogen, Cat#MA5-14657; Mouse anti-NIDOGEN, Santa Cruz Biotechnology, Cat#sc-133175; Goat anti-PODOCALYXIN, R&D, Cat#AF1658; LTL-biotinylated, Vector lab, Cat#B1325; DBA-biotinylated, Vector lab, Cat# B-1035; Mouse anti-EpCAM, CST, Cat#2929S; Rabbit anti-EpCAM, CST, Cat#36746S; Goat anti-SIX2, Proteintech, Cat#11562-1-AP; Mouse anti-SALL1, Perseus Proteomics, Cat#PP-K9814-00; Mouse IgG Isotype Control, Invitrogen, Cat#31903; Normal Goat IgG Control, R&D, Cat#AB-108-C; Donkey anti-Mouse IgG- Alexa Fluor 488, Thermo Fisher Scientific, Cat#A21202; Donkey anti-Rabbit IgG- Alexa Fluor 488, Thermo Fisher Scientific, Cat#A21206; Donkey anti-Goat IgG- Alexa Fluor 488, Thermo Fisher Scientific, Cat#11055; Donkey anti-Guinea pig IgG- Alexa Fluor 488, Jackson ImmunoResearch, Cat#706-545-148; Donkey anti-Mouse IgG- Alexa Fluor 546, Thermo Fisher Scientific, Cat#A10036; Donkey anti-Rabbit IgG- Alexa Fluor 546, Thermo Fisher Scientific, Cat#A10040; Donkey anti-Goat IgG- Alexa Fluor 546, Thermo Fisher Scientific, Cat#A11056; streptavidin, Alexa Fluor 546 conjugate, Thermo Fisher Scientific, Cat#S11225; Donkey anti-Mouse IgG- Alexa Fluor 647, Thermo Fisher Scientific, Cat#A31571; Donkey anti-Rabbit IgG- Alexa Fluor 647, Thermo Fisher Scientific, Cat#A31573; Donkey anti-Goat IgG- Alexa Fluor 647, Thermo Fisher Scientific, Cat#A21447; Hoechst 33342, Thermo Fisher Scientific, Cat#H1399

Validation

Anti-NANOG antibody was validated using undifferentiated human iPS cells (1383D2 cells). The other primary antibodies were validated using human embryonic or adult kidney section samples.

Eukaryotic cell lines

Policy information about cell lines and Sex and Gender in Research

Cell line source(s)

1383D2 human iPSC line, ICSCB, male, iPS cells generated from peripheral blood mononuclear cells of a healthy subject obtained from Cellular Technology Limited; Mild AS patient iPSC line, CiRA, Kyoto University, male, iPS cells generated from peripheral blood mononuclear cells of a patient with Alport syndrome; Mutation-corrected mild AS patient iPSC line, CiRA, Kyoto University, male, iPS cells generated from peripheral blood mononuclear cells of a patient with Alport syndrome; Severe AS patient iPSC line, CiRA, Kyoto University, male, iPS cells generated from peripheral blood mononuclear cells of a patient with Alport syndrome

Authentication

All cell lines were tested for expression of the undifferentiated state marker NANOG, normal karyotype, and multipotent differentiation ability.

Mycoplasma contamination

All cell lines tested negative for mycoplasma contamination.

Commonly misidentified lines
(See ICLAC register)

Name any commonly misidentified cell lines used in the study and provide a rationale for their use.

Animals and other research organisms

Policy information about studies involving animals; ARRIVE guidelines recommended for reporting animal research, and Sex and Gender in Research

Laboratory animals

12-20-week-old female G5X AS model mice (B6.Cg-Col4a5tm1Yseg/J); 10-30-week-old male C57BL/6 mice; 8-12-week-old male NOD/SCID mice

Wild animals

This study did not involve wild animals.

Reporting on sex

Indicate if findings apply to only one sex; describe whether sex was considered in study design, methods used for assigning sex. Provide data disaggregated for sex where this information has been collected in the source data as appropriate; provide overall numbers in this Reporting Summary. Please state if this information has not been collected. Report sex-based analyses where performed, justify reasons for lack of sex-based analysis.

Field-collected samples

This study did not involve samples collected from the field.

Ethics oversight

Animal experiments were approved by the CiRA Animal Experiment Committee and conducted in accordance with institutional guidelines.

Note that full information on the approval of the study protocol must also be provided in the manuscript.

Flow Cytometry

Plots

Confirm that:

- [ ] The axis labels state the marker and fluorochrome used (e.g. CD4-FITC).
- [ ] The axis scales are clearly visible. Include numbers along axes only for bottom left plot of group (a 'group' is an analysis of identical markers).
- [ ] All plots are contour plots with outliers or pseudocolor plots.
- [x] A numerical value for number of cells or percentage (with statistics) is provided.

Methodology

Sample preparation	Kidney organoids were dissociated in TrypLE Select Enzyme (Thermo Fisher Scientific) for approximately 30 min at 37°C. The cell suspension was incubated with primary antibodies in 2% fetal bovine serum (FBS; Wako)/PBS for 30 min on ice. After washing, the cell suspension was incubated with secondary antibodies for 30 min on ice. Antibody-labeled cells were resuspended with 2% FBS/PBS containing 4',6-diamidino-2-phenylindole (DAPI; Sigma).
Instrument	BD FACSAria II
Software	FACS Diva (BD) software program
Cell population abundance	More than 95%
Gating strategy	The cells stained with isotype control and secondary antibodies were used as a negative control. Gating was set such that DAPI(-) live negative control cells had a positive fraction of less than 1%.

☒ Tick this box to confirm that a figure exemplifying the gating strategy is provided in the Supplementary Information.
